# Minimally Invasive Abdominal Repair of a Giant Paraesophageal Hiatal Hernia with Occupation of the Right Thorax in a 53-Year-Old Man

**DOI:** 10.1155/2022/1855656

**Published:** 2022-09-09

**Authors:** Francisco Navarro, Eduardo Pizarro, Marco Ceroni

**Affiliations:** ^1^Oesophago-Gastric Team at Dr. Sótero del Río Hospital, Chile; ^2^School of Medicine, Pontifical Catholic University of Chile, Chile

## Abstract

Paraesophageal giant hiatal hernia is a rare condition associated with serious complications if not treated surgically. There are no reports of the minimally invasive abdominal repair of a giant hiatal hernia of the stomach almost entirely occupying the right thoracic cavity. The most common clinical presentation includes pathological gastroesophageal reflux, dysphagia, chest pain, or respiratory symptoms such as chronic cough or dyspnoea. Chest computed tomography, upper gastrointestinal endoscopy, and high-resolution oesophageal manometry are used to indicate the best treatment. This article reports the minimally invasive abdominal repair of a case of paraesophageal giant hiatal hernia occupying the right thoracic cavity.

## 1. Introduction

Hiatal hernia (HH) is characterized by a protrusion of the stomach into the thoracic cavity through the diaphragmatic hiatus that may also involve another intra-abdominal organ [1]. Most cases are asymptomatic and are diagnosed incidentally by upper gastrointestinal endoscopy or radiological studies. Patients can also present with gastroesophageal reflux, dysphagia, respiratory symptoms, or a condition such as gastric volvulus [2, 3]. Surgical repair is indicated for patients with symptomatic HH and for all patients with paraesophageal HH because of the risk of complications [3]. An HH is considered to be giant when it involves a protrusion of at least 5 cm from the stomach to the thorax or when 30% of the stomach is in the hernial sac [4]. Giant paraesophageal HH accounts for 0.3-15% of all HHs [5].

There are no reports of the repair of an HH occupying the right thoracic cavity with an exclusively minimally invasive approach through the abdomen. The objective of this study is to report a case of the repair of a giant paraesophageal HH occupying the right thoracic cavity involving the stomach and colon with a minimally invasive abdominal approach, without postoperative complications.

## 2. Clinical Case

**Figure 1:**
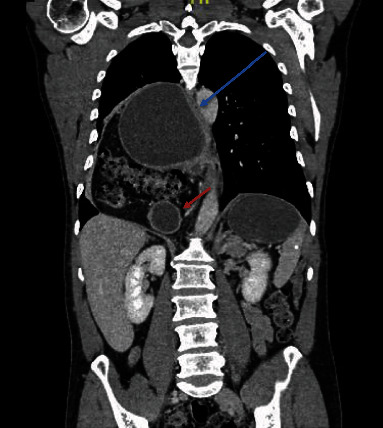
Computed tomography of the thorax, abdomen, and pelvis. Coronal reconstruction showing a giant HH involving the stomach and part of the duodenum and transverse colon. The blue arrow corresponds to the esophagus compressed by the hiatal hernia; the red arrow corresponds to the duodenum.

**Figure 2:**
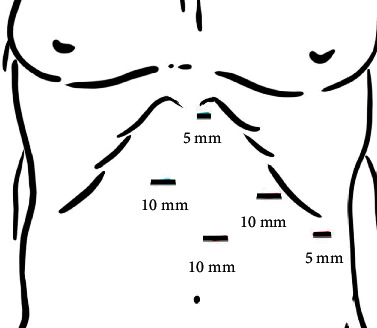
Diagram indicating the position of the trocars. The left and right 10 mm ports are for the surgeon's hands. The supraumbilical port is for the camera. The liver retractor uses the 5 mm subxiphoid incision. Assistant's left hand uses the 5 mm port located on the left flank. Supplementary material (video): https://youtu.be/OMXmnDmL6uU.

**Figure 3:**
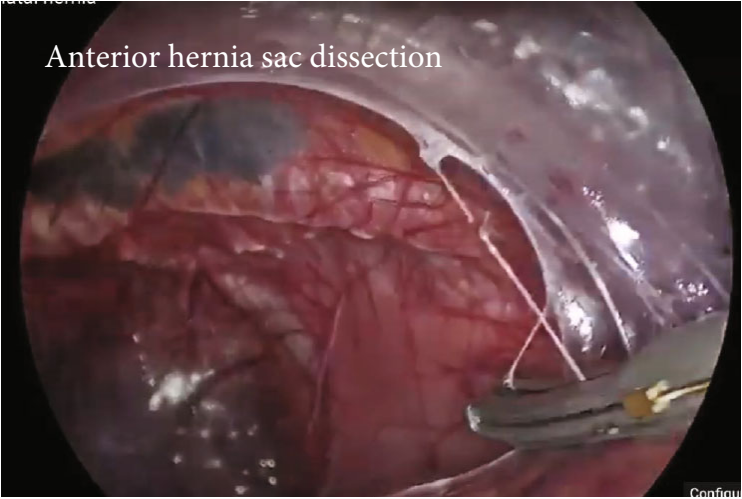
Video screenshot: anterior dissection.

**Figure 4:**
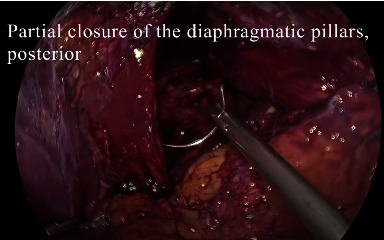
Video screenshot: closure of hiatus.

A healthy 54-year-old male patient had a 3-month history of epigastric pain associated with intermittent vomiting and weight loss of 10 kg. He consulted for upper gastrointestinal bleeding with haematemesis, with hemoglobin of 9 g/dl, which did not require red blood cell transfusions. On admission to the emergency room without hemodynamic compromise, laboratory tests show elevated leukocytes at 11,500 *μ*l and CRP at 50 mg/l (normal value up to 5 mg/l), albumin at 3.5 g/dl, serum potassium 3 mEq/l, and normal arterial gases, without coagulation alteration. Upper gastrointestinal endoscopy reported gastric volvulus with significant anatomical distortion, with mucosal congestion without necrosis, bleeding, or other lesions. Computed tomography of the chest, abdomen, and pelvis with intravenous contrast revealed a giant HH containing a volvulated stomach with edema of its walls without necrosis; in addition, the first two portions of the duodenum and the transverse colon, occupying the right thoracic cavity (Figure 1). High-resolution manometry was not conducted due to the need for emergency surgery. Laparoscopic hiatal hernioplasty was performed with gastropexy of the gastric fundus (Figure 2 shows the position of the trocars; Figures 3 and 4 and video show the surgical technique). The hernial sac was completely dissected through the oesophageal hiatus. The stomach, duodenum, and colon were completely lowered into the abdomen, and the hernial sac was resected. The oesophagus was dissected, yielding an abdominal length of 5 cm. Prior to suturing of the crura, pulmonary recruitment was performed under anaesthesia to achieve complete expansion and tension-free closure of the diaphragmatic crura. A drain was left towards the mediastinum to reduce the risk of developing a mediastinal seroma. Video link https://youtu.be/OMXmnDmL6uU, prior to discharge, upper digestive tract endoscopy was performed with a water-soluble medium, and the findings were normal. The patient was discharged from the hospital on the 5^th^ day without complications. At follow-up, he has not presented symptoms of gastroesophageal reflux, with normal endoscopy and computed tomography, without recurrence of HH.

## 3. Discussion

The gastric volvulus of paraesophageal HH is an emergency pathology that must be resolved quickly, due to the risk of necrosis of the stomach or other organs that are in the hernia sac, which presents a high morbidity and mortality [6–9]. The patient's symptoms, characterized by weight loss, epigastric pain, and upper gastrointestinal bleeding, should lead to suspicion of cancer, so the study in the emergency room should be efficient in order to reach a precise diagnosis. The absence of hemodynamic compromise gave time to perform a computed tomography [7], which reported the presence of a gastric volvulus, without necrosis, but with congestion of the wall and perigastric inflammatory changes, with the organs protruding into the right chest. Upper gastrointestinal endoscopy allows supporting the diagnosis, evaluating the vitality of the stomach; however, due to the anatomical distortion secondary to the gastric volvulus, it was not possible to evaluate the entire gastric mucosa, so the presence of other lesions cannot be ruled out.

Laparoscopy allows a magnified view of the anatomy, so it should be the approach of choice even in emergency patients, if they do not have hemodynamic compromise [10–15]. We routinely use a 30-degree lens in our center for hiatal hernia repairs so as not to interfere with the surgeon's instruments. Regarding the HH treatment technique, we follow the principles recommended in the clinical guidelines: reduction of the content, resection of the sac, dissection of a length of abdominal esophagus of at least 3-6 cm, and closure of the hiatus without tension with nonabsorbable suture [10, 11]. Regarding the use of mesh to reduce the risk of recurrence, our group does not routinely indicate it; we use it only in cases in which it is not possible to close the hiatus without tension. The literature has not shown that mesh consistently reduces the risk of HH recurrence [16–19], and we have seen cases of mesh inclusion in tissues that have required esophagectomy. Regarding the indication of a fundoplication [20], we perform it whenever the manometry rules out a motor disorder of the esophagus. In the present case, because it was an emergency, we did not perform an esophageal manometry.

In the particular case of a HH that occupies the right chest, some precautions must be taken, such as injury to the vena cava in the posterior dissection and the airway in the anterior dissection, which has a more inclined course to the right.

In the use of a drain towards the mediastinum, we routinely indicate in giant HHs, to reduce the risk of developing a mediastinal seroma, which we remove between 5 and 7 days in the postoperative control.

We perform a radiological study on all our patients that demonstrates the absence of recurrence between 1 and 2 postoperative days. In the event of an early recurrence, it can be easily treated laparoscopically.

### 3.1. Lessons from the Case

Gastric volvulus in a giant HH may present with GI bleeding. Upper gastrointestinal endoscopy and computed axial tomography are the fundamental diagnostic studies to have an accurate diagnosis. The laparoscopic approach was useful in emergency HH repair, since it allows a magnified view of the anatomy and avoids injury to other organs.

## Data Availability

Data are available in video link https://youtu.be/OMXmnDmL6uU.
